# Retraction Note: Impact of guidewire selection and operator expertise on radiation exposure in transradial angiography

**DOI:** 10.1186/s13019-015-0251-8

**Published:** 2015-03-27

**Authors:** Jianmin Yang, Ningfu Wang, Xiaoshan Tong, Xianhua Ye, Liang Zhou, Guoxin Tong, Yun Shen, Shuzheng Lv

**Affiliations:** 1 Department of Cardiology, Beijing Anzhen Hospital, Capital Medical University, No.2, Anzhen Road, Beijing, 100029 China; 2 Department of Cardiology, Hangzhou Hospital (Hangzhou No.1 Municipal Hospital), Nanjing Medical University, Hangzhou, China

## Retraction

The Publisher and Editor regretfully retract this article [1] because the peer-review process was inappropriately influenced and compromised. As a result, the scientific integrity of the article cannot be guaranteed. A systematic and detailed investigation suggests that a third party was involved in supplying fabricated details of potential peer reviewers for a large number of manuscripts submitted to different journals. In accordance with recommendations from COPE we have retracted all affected published articles, including this one. It was not possible to determine beyond doubt that the authors of this particular article were aware of any third party attempts to manipulate peer review of their manuscript.
